# Development and Validation of Risk Scores for All-Cause Mortality for a Smartphone-Based “General Health Score” App: Prospective Cohort Study Using the UK Biobank

**DOI:** 10.2196/25655

**Published:** 2021-02-16

**Authors:** Ashley K Clift, Erwann Le Lannou, Christian P Tighe, Sachin S Shah, Matthew Beatty, Arsi Hyvärinen, Stephen J Lane, Tamir Strauss, Devin D Dunn, Jiahe Lu, Mert Aral, Dan Vahdat, Sonia Ponzo, David Plans

**Affiliations:** 1 Department of Surgery and Cancer Imperial College London London United Kingdom; 2 Huma Therapeutics London United Kingdom; 3 Department of Science, Innovation, Technology, and Entrepreneurship University of Exeter Exeter United Kingdom; 4 Department of Experimental Psychology University of Oxford Oxford United Kingdom

**Keywords:** C-Score, mortality, risk score, smartphone, health score, medical informatics, public health, mobile health, development, validation, app, prospective, cohort, machine learning

## Abstract

**Background:**

Given the established links between an individual’s behaviors and lifestyle factors and potentially adverse health outcomes, univariate or simple multivariate health metrics and scores have been developed to quantify general health at a given point in time and estimate risk of negative future outcomes. However, these health metrics may be challenging for widespread use and are unlikely to be successful at capturing the broader determinants of health in the general population. Hence, there is a need for a multidimensional yet widely employable and accessible way to obtain a comprehensive health metric.

**Objective:**

The objective of the study was to develop and validate a novel, easily interpretable, points-based health score (“C-Score”) derived from metrics measurable using smartphone components and iterations thereof that utilize statistical modeling and machine learning (ML) approaches.

**Methods:**

A literature review was conducted to identify relevant predictor variables for inclusion in the first iteration of a points-based model. This was followed by a prospective cohort study in a UK Biobank population for the purposes of validating the C-Score and developing and comparatively validating variations of the score using statistical and ML models to assess the balance between expediency and ease of interpretability and model complexity. Primary and secondary outcome measures were discrimination of a points-based score for all-cause mortality within 10 years (Harrell c-statistic) and discrimination and calibration of Cox proportional hazards models and ML models that incorporate C-Score values (or raw data inputs) and other predictors to predict the risk of all-cause mortality within 10 years.

**Results:**

The study cohort comprised 420,560 individuals. During a cohort follow-up of 4,526,452 person-years, there were 16,188 deaths from any cause (3.85%). The points-based model had good discrimination (c-statistic=0.66). There was a 31% relative reduction in risk of all-cause mortality per decile of increasing C-Score (hazard ratio of 0.69, 95% CI 0.663-0.675). A Cox model integrating age and C-Score had improved discrimination (8 percentage points; c-statistic=0.74) and good calibration. ML approaches did not offer improved discrimination over statistical modeling.

**Conclusions:**

The novel health metric (“C-Score”) has good predictive capabilities for all-cause mortality within 10 years. Embedding the C-Score within a smartphone app may represent a useful tool for democratized, individualized health risk prediction. A simple Cox model using C-Score and age balances parsimony and accuracy of risk predictions and could be used to produce absolute risk estimations for app users.

## Introduction

### Background

Despite the empirical establishment of strong relationships between given behaviors and lifestyle factors and the development of preventable diseases, individuals may struggle to tangibly conceptualize how their day-to-day behavior affects their long-term health outcomes. A number of mortality risk algorithms or “health metrics” have been developed to quantify general health at a given point in time and estimate risk of negative future outcomes; however, few of these tools are accessible, interpretable, actionable, and easy to calculate [1-3]. Furthermore, their degrees of validation differ [2,3]. The use of univariate measures, while easily calculable and interpretable, may incompletely capture the wider determinants of health, such as psychological well-being.

BMI is often used as a quick means of estimating an individual’s relative adiposity and infer the relative likelihood of adverse adiposity-related outcomes [4,5]. Despite its relative ease of calculation, BMI has numerous oft-promulgated limitations, including issues with scalability (two people with the same body proportions but different heights will have divergent BMIs); its ignorance of variation in physical characteristics due to age, sex, or ethnicity [6,7]; its inability to discern between muscle and fat; and its variable strength of relationship to health outcomes [8].  

Multivariable risk prediction models can be easily developed using statistical modeling [9,10] or machine learning (ML) [2,11,12] approaches on appropriate data sets and lend themselves to supporting decision making across the manifold aspects of health and disease management or prevention. Indeed, there is a multitude of models published that seek to predict all-cause mortality [1,2]. All-cause mortality is an easily understandable risk trajectory into which the natural histories of many preventable diseases converge and can be manipulated by behavior changes. Therefore, it represents an attractive target for a general health metric or predictive model. However, there are hurdles to the widespread use of such predictive models [13]. Validation of models in the data sets that they were derived from (internal validation) and an assessment of their ability to generalize to independent data sets—preferably in different populations (external validation)—must be achieved prior to widespread use [14,15]. However, even when validated, models tend to remain the preserve of clinicians and may incorporate mathematical analysis of data points that require invasive testing (eg, blood tests), may be nonmodifiable by users (eg, childhood exposures), or are not easily accessible (eg, Townsend deprivation index).

Therefore, an unmet need in public health is the presence of validated health metrics based on models that are not only strongly predictive of outcomes but also accessible, have an understandable/interpretable output, and are parsimonious. Furthermore, should causal mechanisms be clearly established and the metrics validated as “causal prediction models,” the focused use of modifiable predictor variables could help demonstrate actionable insights to guide beneficial lifestyle changes. Given the ability of smartphones to utilize inbuilt hardware to capture multimodal data relevant to physiological status, we believe that a smartphone app integrating product design and technological and risk modeling principles could present a novel conduit for risk prediction models focusing on well-established risk factors to enable members of the general public to engage with their health.

Here, the authors describe the development of a novel multivariable health metric, hereon named “C-Score”, which seeks to mathematically integrate parameters that can be measured digitally, are almost all modifiable, and are relevant to various domains of health. Three formats of risk score or model were developed: (1) a simple, easy-to-interpret, 0-100 points–based score developed by summation of published literature regarding multiple variables across multiple geographic locations; (2) statistical modeling using Cox proportional hazards methods analyzing C-Score with other predictors such as age; and (3) ML models analyzing C-Score and the same predictor variables as used for statistical modeling. The first was validated, and the latter two were developed and validated using the UK Biobank [16] data.

### Objective

This study sought to develop and validate forms of novel risk models for the purposes of a general health metric suitable for embedding into a smartphone app. Given the convergence of multiple key risk factors on the risk of all-cause mortality, as well as morbidity and mortality from leading noncommunicable diseases, the target endpoint chosen for this metric was all-cause mortality.

## Methods

The study was planned and conducted in accordance with TRIPOD guidelines [17].

### Candidate Explanatory Variables for Models

A comprehensive literature review was conducted using PubMed for candidate predictor variables for all-cause mortality. Search terms included “all-cause mortality,” “death,” “mortality prediction,” and “risk model.” In addition, preposited candidate variables were searched alongside “all-cause mortality,” such as “smoking AND all-cause mortality” and “resting heart rate AND all-cause mortality.”

The candidate variables that were identified from the literature review, which was led by clinical and epidemiological acumen regarding biological plausibility, were considered by the authorship panel in terms of their evidence base. They were also considered in terms of the degree to which they are modifiable, their ability to be measured using inbuilt capabilities of commonly available smartphones, and their contributions to engaging user design perspectives. As the intention was to develop an interpretable “general health metric” generated using an explainable underlying model that would be relevant to multiple morbidities rather than mortality alone, candidate variables were reviewed in terms of overlap with leading causes of morbidity and mortality.

Ultimately, eight predictor variables were selected: age, cigarette smoking, alcohol intake, self-rated health, resting heart rate, sleep, cognition (reaction time), and anthropometrics (waist-to-height ratio).

### Development of a Points-Based Score (C-Score) 

The first risk index (“C-Score”) attempted to formulate an easy-to-interpret continuous score that used published evidence from multiple countries focused chiefly on modifiable factors, as opposed to developing a model using a single large database from one geographic location. This approach sought to utilize published hazard ratios or regression coefficients to weight individual parameters, as has been done elsewhere [18,19].

Studies identified using the above search criteria were reviewed by the authorship panel for cohort size, length of follow-up, robustness of statistical analysis, and whether or not hazard ratios for all-cause mortality were reported (and if these were adjusted for age, gender, and/or other confounders).

We opted to include all important variables other than age in the first iteration of the points-based model to ascertain the power of purely dynamic/modifiable characteristics in a risk index.

Hazard ratios were extracted from the studies deemed to be of the highest quality by the authorship panel. These were used as relative “weightings” for a points-based score. The optimal value for each input was set as 0 (lowest risk), with increasing numbers of points assigned for greater diversions away from optimal risk level (these points reflected the literature-extracted hazard ratios). The raw sum of maximal hazard ratios was approximately 25; therefore, the values for all increments of hazard ratios were quadrupled to make a total sum of 100.

The points-based score functions in a penalizing fashion—that is, users “start” with 100 points, and for each health domain, they can sequentially either lose no points (if they optimize that data input) or lose points in accordance with the hazard ratio associated with that level of exposure. For example, users will lose no points for being a never-smoker but will lose more points if they smoke more than 20 cigarettes per day than if they smoke 10 cigarettes per day. As such, the output from the score is a continuous variable—a number between 0 and 100, where 100 is optimal. Table 1 demonstrates the maximum penalty for each subdomain, as informed by extracted hazard ratio data [1,20-25].

**Table 1:** The range of points allocated to users per data input to C-Score.

C-Score input metric	Domain	Range of points allocated
Resting heart rate (beats per minute)	Cardiovascular fitness	0-7.83
Average hours of sleep per night	Sleep habits	0-10.26
Waist-to-height ratio	Adiposity	0-10.80
Self-rated health (ordinal scale: excellent, good, fair, poor)	Surrogate for existing comorbidity or perception of ill health	0-31.32
Cigarette smoking (status and cigarettes per day)	Tobacco exposure, including past smoking	0-12.96
Alcohol consumption (units/week)	Alcohol intake	0-19.44
Reaction time (ms)	Neurocognition	0-6.75

As the C-Score does not output a percentage risk prediction, it was only assessed for discrimination in predicting all-cause mortality within 10 years. As is evident below, percentage absolute risk assessments are possible if the C-Score is incorporated as a variable in statistical modeling approaches.

### App Data Collection Methods and Materials

**Figure 1:**
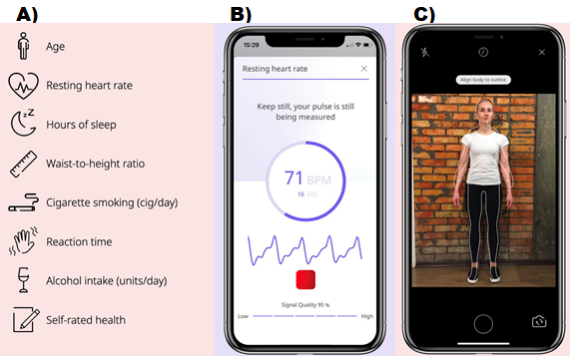
Screenshots of the C-Score mobile app. (A) List of data contributing to the C-Score. (B) Resting heart rate measurement screen. (C) Body scan screen (for waist-to-height ratio).

The raw data points contributing to the C-Score calculation are intended to be obtained using an ad hoc smartphone app (Figure 1). Data inputs for the score include manual entry (eg, age, gender, and alcohol and tobacco consumption), image analyses (eg, waist-to-height ratio), use of phone camera technology (eg, for resting heart rate), and screen-based reaction time testing. Waist-to-height ratio was calculated using camera-based anthropometric measurements (body volume index), which produced the following outputs: waist circumference, hip circumference, body fat percentage, and total body volume. Resting heart rate was collected via a camera-driven photoplethysmogram sensor, which is able to detect the heartbeat when participants position their finger on the camera. Reaction time in the app is measured by asking users to hold their finger on the screen and lift it when the screen changes color; users are asked to repeat the test three times and the average time across the trials is then computed.

### Study Population

The individual-level data of the UK Biobank were utilized as the study population for the validation of the points-based model and the development and validation of risk models for all-cause mortality that incorporate C-Score inputs (as more complex variants of the initial points-based score). The entirety of the available data set with complete data regarding C-Score inputs was used for our analyses. Briefly, the UK Biobank represents a prospective cohort study where over 500,000 individuals aged 40 to 69 years were recruited between 2006-2010 and followed up thereafter [16]. Participants underwent a robust assessment at baseline, during which multiple questionnaires were completed, anthropometric and other measures were taken, and blood and saliva samples were obtained. In addition to robust phenotyping, all participants have been genotyped, 100,000 are in the process of undergoing whole-body imaging, and 20,000 have completed repeat assessments. Participants’ data are linked to multiple other electronic databases, enabling the ascertainment of data regarding date and cause of death. The UK Biobank is an “open” resource accessible to any researcher approved as bona fide by the UK Biobank Access Management Team.

### Development of De Novo Models

Whereas C-Score was validated in terms of discrimination using the UK Biobank data, four additional model versions were both generated and validated using data from the UK Biobank, referred to as models 2 to 5. As the continuous “health score” does not predict percentage risks, we used Cox regression to form variants of models that can output such a risk prediction and assess their discrimination and calibration. This opens the possibility of having a user-facing score, with scope for generating individualized percentage-style risk estimates for multiple outcomes of interest, such as all-cause mortality.

Model 2 integrated C-Score and age, whereas model 3 integrated the raw values for all C-Score inputs and age to assess the amount of performance sacrificed by a predetermined weighting system. Model 4 sought to develop “maximally complex” statistical models with interactions to identify the maximum attainable predictive accuracy and also included sex and ethnicity, again to assess the balance between predictive power and expediency of a simple, interpretable score or simple model. These Cox models were developed to predict the risk of death within 10 years of follow-up as a complete case analysis. As the intended smartphone app would require completion of all data fields to generate the health score, a complete case analysis of UK Biobank data offered a form of evaluation that most closely aligned to the intended use of the models. The baseline data values (ie, obtained from the assessment center) were used to calculate C-Scores and also participants’ baseline age for the development of the Cox models. Individual follow-up was defined as time elapsed from initial assessment to either death from any cause or censoring (lost to follow-up or reached the end of study date). The end of study date was set as February 9, 2020, which corresponded to the date of data extract download.

### Development of ML-Based Models

The approach taken for the development of model 5 was to use supervised ML. The problem was specified as a binary classifier, aiming to assign a label representing whether or not the patient dies 10 years postbaseline. Two commonly used supervised ML classifiers were chosen, the K-nearest neighbor (KNN) classifier and the support vector machine (SVM) classifier. As the C-Score was conceptualized as a user-friendly, easily explainable metric, we chose to assess KNN and SVM modeling approaches because their mechanistic underpinnings can be relatively easily relayed to a user, compared with, for example, a neural network or boosting algorithm. Both these algorithms were tuned to select the optimal hyperparameters using 10-fold cross-validation on the training and validation sets (to maximize the area under the receiver operating characteristic curve [AUROC]).

In the UK Biobank cohort, the number of occurrences of the outcome of interest was relatively low (less than 5%). During the SVM and KNN development and evaluation, it became apparent that the outcome sparsity might have had implications for model performance (with initial AUROCs ranging between 0.67 and 0.68 when using a 70:30 “split”). The final model was trained by randomly undersampling the training data (in order to achieve a 50:50 split between the two outcome variables). It is also important to note that these two algorithms are based in feature space, so the weighting of each feature plays a crucial role in the determination of the classification coefficients. As such, it is important to standardize all of the inputs; this was performed by first subtracting the mean and subsequently dividing by the standard deviation for each feature.

We first developed and trained a KNN algorithm to derive a binary label determining the patient’s risk of death in the next 10 years. KNNs are a type of classification algorithm based on the premise that similar cases (in feature space) will have similar results. The idea is to classify each new observation based on a metric of “nearness” to all other points and to set its label as the most common label of the K most similar training examples. To use the KNN algorithm, two hyperparameters need to be specified: (1) the value of K (ie, how many training examples will it aggregate to determine the label of the test), and (2) the metric for defining “nearness.” For both of these parameters, we tuned our model using 10-fold cross-validation.

The hyperparameters used for defining “nearness” are the two most commonly used distance metrics, namely the Euclidean distance and the Manhattan distance. The other parameter to be tuned was the value of K—values between 5 and 500 were tested. The optimal parameters were determined by maximizing the AUROC using 10-fold cross-validation.

We trained an SVM classifier to optimally separate in feature space between patients. SVMs are a category of classifiers that aim to determine the hyperplane that optimally separates the observations into two sets of data points. The intuitive idea behind the SVM algorithm is to maximize the probability of making a correct prediction by determining the boundary that is the furthest from all of the observations.

Similar to the previous KNN model, considerations in training were taken into account in choosing the hyperparameters. In the case of SVMs, the parameters we chose to optimize were the shape of the separation kernel (linear, polynomial, or radial basis function [RBF]), the C regularization parameter, the degree of the polynomial (only true for polynomial kernels), and the gamma kernel coefficient (for polynomial and RBF kernels). To optimize these parameters, we used 10-fold cross-validation on the training data to maximize the AUROC.

### Statistical Analyses and Model Validation

Continuous variables for descriptive statistics are presented as means and standard deviations. Cox models were developed using the entire available data set and then underwent internal validation using bootstrapping with 200 iterations (for discrimination and calibration). Models were tested for proportional hazards assumptions (using log-log plots) and inclusion of restricted cubic splines or logarithms for continuous variables.

Discrimination refers to the ability of a prediction model to distinguish between individuals that experience an outcome of interest and those who do not. Suitable metrics include Harrell c-statistic, which is equivalent to the AUROC for Cox models. A value of 0.5 means that the model is no better than tossing a coin, whereas a value of 1 means perfect prediction.

Calibration refers to the assessment of closeness between predicted and observed risks. This can be assessed by plotting the observed and predicted risks across different levels, such as by tenth of risk. However, “binning” of risk levels is not optimal, and other approaches include linear adaptive spline hazard regression, which interpolates across levels of risk [26]. Therefore, we assessed calibration of the models using smoothed calibration plots to compare predicted and observed risks, which also incorporated bootstrapping to correct for model optimism.

Bootstrap optimism-corrected values for the c-statistic were computed, and calibration plots were formed for models 2 to 4. Initial data handling was performed using Stata v16.0 software (StataCorp LLC), with the statistical analyses handled in R statistical software, notably the rms package. For model 5, algorithms were developed using Python, including the Pandas, NumPy, and Scikit-learn packages; the AUROC is presented.

### Ethical Approval

Access to anonymized data for the UK Biobank cohort was granted by the UK Biobank Access Management Team (application number 55668). Ethical approval was granted by the national research ethics committee (REC 16/NW/0274) for the overall UK Biobank cohort.

## Results

### Study Population Characteristics

In the complete case analysis, there were 420,560 individuals with complete data, including age at baseline assessment and all metrics included in the C-Score. There was a maximum follow-up of 13.9 years, and the total follow-up time for the cohort was 4,526,452 person-years. During this period, there were 16,188 deaths (3.85% of the cohort).

Demographics for the study cohort were as follows: mean age at baseline was 56.58 (SD 8.07) years, mean resting heart rate was 69.84 (SD 11.68) beats/minute, mean waist-to-height ratio was 0.54 (SD 0.075), mean weekly alcohol intake was 14.34 (SD 18.84) units, mean reaction time was 558.03 (SD 117.07) ms, and mean sleep duration was 7.16 (SD 1.26) hours. For self-rated health, 68,926 (16.39%) subjects reported “excellent,” whereas 245,171 (58.30%), 88,195 (20.97%), and 18,268 (4.34%) subjects reported “good,” “fair,” and “poor,” respectively. There were 230,798 men (55.14%) and 188,601 women (44.86%). Regarding ethnic background, subjects were categorized as “White” (397,763, 94.92%), “mixed” (2480, 0.59%), “Asian or Asian British” (7631, 1.82%), “Black or Black British” (6370, 1.52%), “Chinese” (1293, 0.31%), or “Other” (3524, 0.84%).

**Figure 2:**
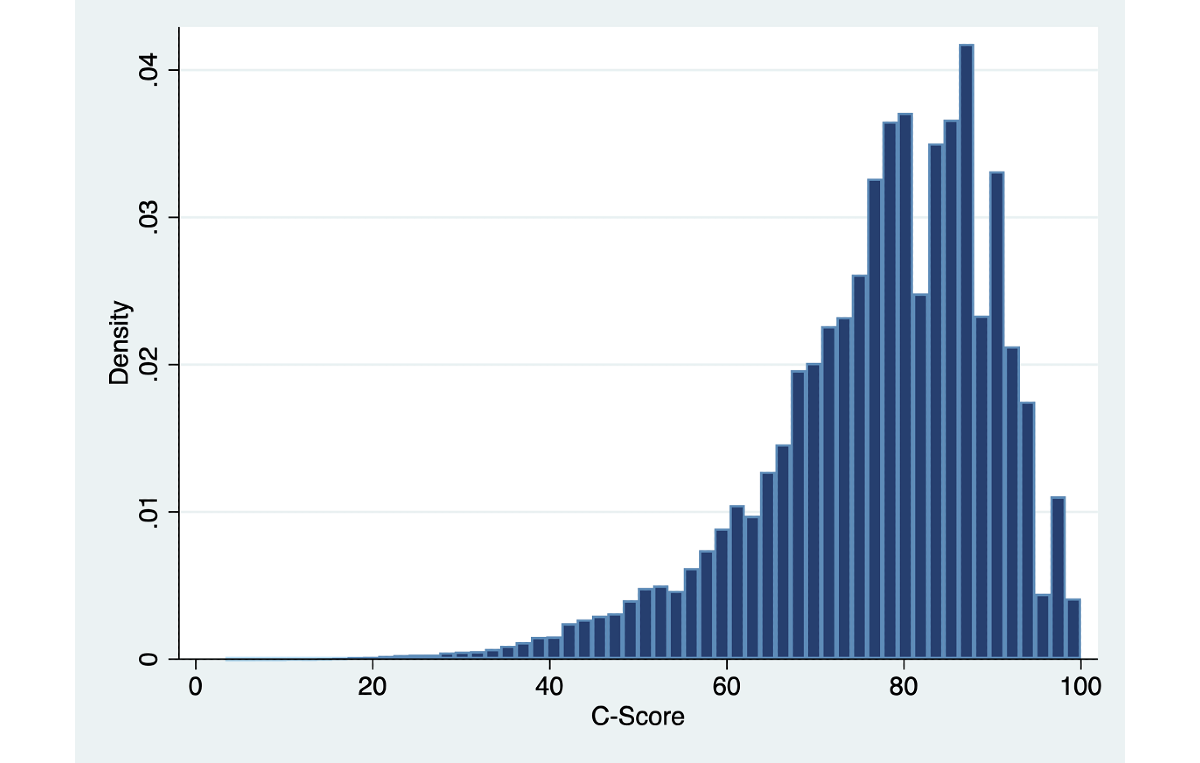
Distribution of C-Score values in the study cohort (N=420,560).

**Figure 3:**
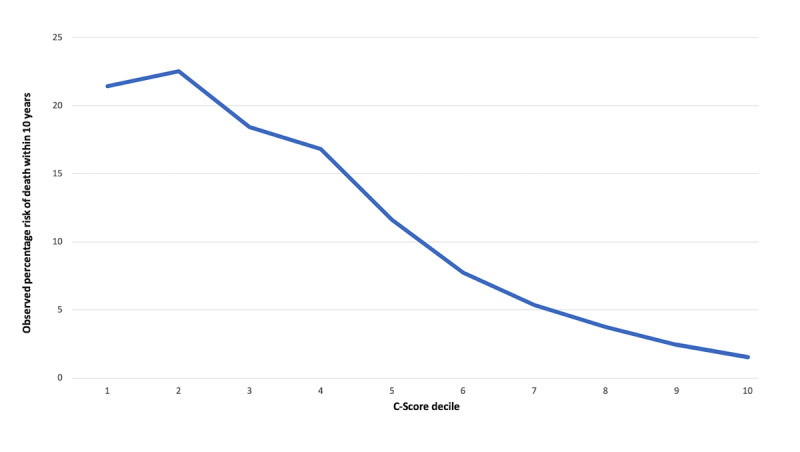
Probability of death within the next 10 years as a function of C-Score decile.

Regarding calculated C-Scores, the mean score for participants was 77.25 (SD 12.96; minimum 3.34, maximum 100; Figure 2). Figure 3 displays the risk of death within 10 years according to C-Score decile.

### Discrimination and Calibration

#### Model 1

The hazard ratio for per-unit increase in C-Score was 0.96 (95% CI 0.960-0.961), suggesting a 4% relative risk reduction per unit improvement. When analyzed in terms of C-Score decile (ie, 10-point brackets of C-Scores), the hazard ratio was 0.69 (95% CI 0.663-0.675), implying a 31% relative risk reduction of all-cause mortality per decile improvement in C-Score. Regarding discrimination, the c-statistic was 0.66.

#### Model 2

**Figure 4:**
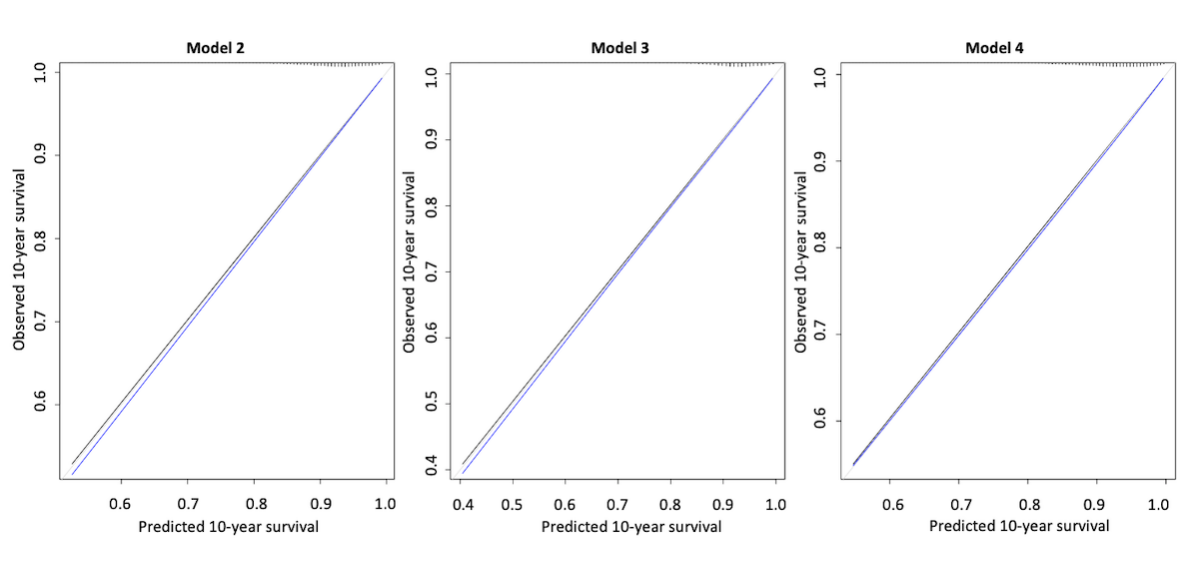
Calibration plots of predicted versus observed probabilities of all-cause mortality within 10 years for models 2, 3, and 4. The ticks across the upper plot border represent the distribution of predicted risks in the study cohort population. The black line displays apparent calibration and the blue line displays the bias-corrected calibration.

Inclusion of (log)age and C-Score in a Cox model yielded a c-statistic of 0.74 (ie, an increase in discrimination capability of 8 percentage points). The model appeared well-calibrated (Figure 4). Although age is clearly a major predictor of all-cause mortality, the Cox model demonstrated that on the inclusion of age and C-Score, there were significant roles for both: hazard ratio per year increase in age was 1.09 (95% CI 1.091-1.096) and per 10-unit increase in C-Score was 0.67 (95% CI 0.668-0.681). Table 2 demonstrates the coefficients for all Cox models developed.

**Table 2:** Coefficients from Cox proportional hazards models either examining C-Score alone or alongside additional parameters/interactions or raw data inputs (model 3).

Model and predictor variables				Coefficient (*P* value)		Discrimination (c-statistic)^a^
**Cox model with C-Score as sole variate**						0.66
	C-Score		–.0402 (<.001)			
**Model 2**						0.74
	C-Score		–0.0393 (<.001)			
	(log)age		5.0965 (<.001)			
**Model 3**						0.74
	(log)age		3.8622 (<.001)			
	Sleep hours		0.0750 (<.001)			
	**Self-rated health**					
		Good	0.0964 (.01)			
		Fair	0.6310 (<.001)			
		Poor	2.5479 (<.001)			
	Cigarettes smoked per day		0.0685 (<.001)			
	Reaction time		0.0008 (<.001)			
	Waist-to-height ratio		1.1679 (<.001)			
	Weekly alcohol units		0.0077 (<.001)			
	Resting heart rate		0.0133 (<.001)			
**Model 4^b^**						0.74
	C-Score		–0.0874 (<.001)			
	(log)age		4.1934 (<.001)			
	**Ethnic group**					
		Mixed	–0.6416 (.29)			
		Asian/Asian British	0.3550 (.25)			
		Black/Black British	–0.2142 (.61)			
		Chinese	0.1931 (.88)			
		Other	–0.8077 (.10)			
	Male sex		0.2132 (.006)			
	**C-Score^c^**					
		(log)age	0.0121 (.006)			
		Mixed ethnicity	0.0067 (.44)			
		Asian/Asian British ethnicity	–0.0094 (.04)			
		Black/Black British ethnicity	–0.0005 (.93)			
		Chinese ethnicity	–0.0060 (.72)			
		“Other” ethnicity	0.0122 (.10)			
		Male sex	0.0010 (.33)			

^a^For reference in terms of discrimination, a Cox model using simply the C-Score as the sole variate was fitted to demonstrate the scope for incremental gains in accuracy. For models 3 and 4, the coefficients for the “excellent” self-rated health and White ethnicity are the reference categories, and therefore their coefficients equal 0.

^b^Model 4 is presented prior to backward selection.

^c^Denotes interaction terms.

#### Model 3

Using the raw data inputs rather than a preassigned “weighting” plus age yielded a c-statistic of 0.74; therefore, there was no significant improvement in discrimination with a more complex model. The model was also well-calibrated (Figure 4).

#### Model 4

We also developed a “full model” that included C-Score, age, ethnicity, and sex, as well as interactions between C-Score and the latter three variables. We performed backward elimination to identify the strongest possible performing model via bootstrapping with 200 iterations; selection was based on the Akaike information criterion with a *P* value of 0.01. Herein, the final model that retained C-Score, sex, age, an interaction between C-Score and ethnicity, and an interaction between C-Score and age had an optimism-corrected c-statistic of 0.74 (ie, no improvement with a more complex model).

#### Model 5

Both of the ML algorithms, KNN and SVM, were applied to a test cohort (n=125,966) in order to predict risk of death in the next 10 years. For the KNN, following 10-fold cross-validation for the tuning of the hyperparameters (we opted to use K=100 and the Manhattan distance metric), the c-statistic on the test data was 0.72. Similar to the KNN algorithm, we tuned the SVM using 10-fold cross-validation on randomly under-sampled training data. This led us to choose C=100, gamma=0.001, and kernel shape as an RBF as the optimal hyperparameters.

## Discussion

### Principal Findings

Risk prediction models have significant potential for assessing the risk of protean events of interest. However, these models are limited almost exclusively to clinical use, and widely used/easily accessible health metrics, such as BMI, have limitations. Extant multivariable prediction models for all-cause mortality are typically poorly accessible to members of the public and risk limited engagement due to perceived nonmodifiability of covariates or limited ability to understand the mechanisms by which covariates may predict outcomes. Therefore, for the purposes of this initiative, we opted to migrate away from univariate assessments toward a novel, multivariable health metric that is focused on characteristics that span multiple domains of health, is accessible, and can be used by anybody with a smartphone. Our results demonstrate the value of an easy-to-interpret points-based score to infer all-cause mortality risk and mandate consideration of this smartphone-based health index in the prediction of multiple other diseases or conditions. Our results also suggest that a simple Cox model including C-Score and age may provide accurate absolute risk predictions for public health initiatives, such as promoting public understanding of individual health risk or raising awareness of the effects of behaviors on health. Lastly, the results are interesting regarding the power of statistical modeling approaches relative to ML approaches using the same data.

All-cause mortality was selected as a first end point for validation purposes given its ease of comprehension and its close links to multiple modifiable behaviors and/or it often being a consequence of preventable disease. This is an end point that has been robustly examined in the UK Biobank by two other key studies. A study by Weng et al [2] utilized the UK Biobank to derive epidemiological models (ie, Cox) and ML models (ie, random forests and neural networks) to predict premature mortality using a preselected panel of 60 candidate baseline predictor variables encompassing aspects such as sociodemographics, educational attainment, behavior, nutritional intake, lifestyle factors, medication use, and clinical history. In standard Cox modeling, the final included variables were gender; log(age); educational qualifications; ethnicity; previous diagnoses of cancer, coronary heart disease, type 2 diabetes, or chronic obstructive pulmonary disease; smoking history; blood pressure; Townsend deprivation index; and BMI. Variables included in random forests modeling included BMI, forced expiratory volume in the first second of expiration, waist circumference, blood pressure parameters, skin tone, and age. On identifying the optimal neural network parameters using grid-search from 10-fold cross-validation, top risk factor variables included smoking status, age, prior cancer diagnosis, prescription of digoxin, residential air pollution, and Townsend deprivation index. The discrimination of the fully adjusted Cox, random forests, and neural network models were 0.751 (95% CI 0.748-0.767), 0.783 (95% CI 0.776-0.791), and 0.79 (95% CI 0.783-0.797), respectively [10]. While these AUROC values are significantly but marginally higher than those reported with our intended app-based model, they included variables that are, for the most part, nonmodifiable and do not offer clear scope for use by members of the public to not only compute their risk but also be able to act on various components to reduce risk.

Ganna and Ingelsson [1] used the UK Biobank cohort to identify predictors of 5-year all-cause mortality and six cause-specific mortality categories from 655 measurements of demographic variables, the results of which were interestingly packaged as part of an interactive website named Ubble. Ultimately, for all-cause mortality within 5 years, 13 predictors for men and 11 for women in a Cox model achieved discrimination of 0.8 (95% CI 0.77-0.83) and 0.79 (95% CI 0.76-0.83), respectively. Again, although these models attained a significantly but slightly higher discrimination than our model, the majority of parameters included are minimally modifiable (in retrospect, number of children given birth to), have an effect on mortality that is difficult to explain (number of people in the household, numbers of cars or vans owned/used by the household, relationships of people lived with), and emphasize existing health conditions (known diabetic, previous cancer) [1]. While our study did not validate the C-Score as a “causal prediction model,” where coefficients have a direct causal interpretation, such further work is underway, and the inclusion of modifiable factors known to have causal implications on health outcomes is encouraging in this regard.

In the era of “big data,” a resurgence in the popularity of artificial intelligence and more specifically ML has been seen across a wide array of fields including health care. These novel methodologies have led to some notable results in prediction and diagnostics and so have become a commonly examined tool in medical research. It is, however, important to note that ML techniques do not always lead to better results than “classical” statistical methods. Indeed, the results that we observed using two very popular and widely used algorithms, namely KNN and SVM, were comparatively similar or even lower than the results observed using a traditional epidemiological/statistical modeling strategy. ML methodologies rely on the artificial generation of knowledge using machine-guided computational methods instead of human-guided data analysis in order to find a best fit in the data. There are some very strong cases for their use, especially when dealing with wide and complex data sets with multifactorial causation and complex and potentially nonintuitive interactions. However, in this study, we showed that ML is not always the answer and that initial development of an algorithm with few metrics and careful consideration of the input by those with scientific/clinical acumen can yield better results.

### Strengths and Limitations

Our work has some strengths and also limitations. Strengths include the use of the UK Biobank, which provided a contemporary, richly phenotyped cohort with linkages to national registries that minimized loss to follow-up, prospectively evaluated risk factors, and enabled accurate ascertainment of outcomes of interest. Another strength was our cognizance of the target users of the app that the model was intended for, which drove us to focus on modifiable risk variables where possible—we were content with sacrificing a small percentage of discriminatory capability without needing to “penalize” intended users for having pre-existing conditions or a certain educational level, or living in areas of heavy air pollution.

Possible limitations of our work include the use of a complete case analysis, which may have introduced bias, and the use of “human intelligence” to prune the possible list of candidate predictor variables, which could have limited the scope for ML to perform optimally. As the overwhelming majority of missing data for the included variables were due to participants “not knowing” the answer or refusing to answer, we considered this to replicate the target end situation, where people will be using the health index or model in an app. We mitigated bias to the best of our abilities throughout the rest of the methodology for the statistical modeling where possible—for example, we used the entire data set for Cox modeling and bootstrapping for validation rather than randomly splitting the data into development and validation sets, which is inefficient and inadvisable [10]. The fact that ML methods did not deliver significant improvements in discrimination is not a formal comparative assessment of statistics versus ML. Indeed, ML is likely best reserved for situations other than trying to optimize the weighting of a small number of variables that humans have preselected, or for situations in which model explainability is less crucial. The validation schemas were different between the approaches, with resampling validation used in statistical modeling and a train-test split used to tune hyperparameters and then assess performance of the ML classifiers. Using the same cross-validation for both hyperparameter tuning and performance assessment on the entire data set is inherently optimistically biased, and while nested cross-validation may be one approach to using all of the data for training and validation, we were unable to do it because of computational limitations. There were also the limitations of data availability and potential selection bias concerning the participants of the UK Biobank. In terms of data availability, the reaction time used at the UK Biobank baseline assessments (2006-2010) was not exactly the same as the reaction test developed for the app: results from a study using the NHANES cohort [23] were used to develop the initial score and inform the reaction time test in the app. However, because the underlying way in which points are allocated for reaction time are based on the relative distribution of time measurements, applying exactly the same cutoff principles (based on standard deviations from the mean) was a pragmatic and suitable way to validate the C-Score in a cohort with a different measurement mechanism. In terms of UK Biobank participants, they tend to be slightly healthier than the general population at large [16,27]. Furthermore, the UK Biobank only recruited individuals between the ages of 40 and 69 years who were generally more affluent and more likely to be of White ethnicity than the general population. Use of the C-Score outside the UK population and in different age groups should follow validation in appropriate local data sets with cognizance of the need for performance evaluation in different ethnic groups, work on which is underway.

### Conclusion

In conclusion, we believe that the “general health metric” reported here not only compares well to other work despite using fewer variables but offers several advantages from a population-use perspective, as it offers a holistic review of multiple aspects of health and focuses for the most part on modifiable characteristics that could in time be targets for risk-reducing interventions pending further model evaluation. Our proclivity was not to produce the most powerfully predictive models possible using a prospective data set but rather to develop and validate models that are rational, understandable, and could be engaging within a smartphone app. Given the strong association of many of the included variables on other diseases (and not just all-cause mortality), we believe that a points-based score may be powerful in making inferences regarding current and future health in terms of individual conditions. Even more powerful could be simple statistical models incorporating C-Score and age for each clinical end point of interest. Further work is already underway within our group to assess the capabilities of C-Score and variations thereof across a panel of conditions of interest, as is the embedding of this score system into a mobile app.

## Abbreviations

AUROC: area under the receiver operating characteristic curve
KNN: K-nearest neighbor
ML: machine learning
RBF: radial basis function
SVM: support vector machine
